# Factors Associated With Aging Biomarkers: Findings From the National Health and Aging Trends Study

**DOI:** 10.1093/geroni/igaa057.1973

**Published:** 2020-12-16

**Authors:** Laura Samuel, Thomas K M Cudjoe, Eileen Crimmins

## Abstract

Few studies have examined associations between socioeconomic, psychosocial and environmental characteristics with biological markers of aging among nationally representative older adult samples. This symposium will present results from four studies that examine the associations between 1) socioeconomic factors (i.e. financial strain and income to poverty ratio), 2) environmental characteristics (i.e. home disorder, street block disorder and community social cohesion) 3) social isolation (i.e. household size and social network),and 4) subjective well-being (i.e. positive affect, self-realization and personal mastery) as they relate to biomarkers of aging (hemoglobin A1c, IL-6, high-sensitivity CRP, and cytomegalovirus). Biomarker samples were obtained in 2017 via dried blood spots from 4,648 (88%) of the 5,265 self-responding participants of the National Health and Aging Trends Study (NHATS). NHATS is an ongoing study that conducts annual in-home interviews, which recruited a nationally representative cohort of Medicare beneficiaries aged 65+ residing in the contiguous United States in 2011 and replenished the sample in 2015. All analyses for the four studies presented in this symposium adjusted for demographic and socioeconomic characteristics and other potential confounders. Sampling weights were applied to account for study design and non-response so that inferences can be generalized to US adults aged ≥67 in 2017. Sessions of this symposium will highlight the socioeconomic, psychosocial and environmental characteristics that are associated with aging biomarkers. These results have clinical, policy and public health implications. These results can inform the development of interventions and policies aimed at improving biologic aging across the lifespan and reducing disparities in biologic aging.

